# Effect of a Virtual Reality Contact-Based Educational Intervention on the Public Stigma of Depression: Randomized Controlled Pilot Study

**DOI:** 10.2196/28072

**Published:** 2022-05-02

**Authors:** Wey Guan Lem, Ayako Kohyama-Koganeya, Toki Saito, Hiroshi Oyama

**Affiliations:** 1 Department of Clinical Information Engineering Graduate School of Medicine The University of Tokyo Tokyo Japan

**Keywords:** major depressive disorder, depression stigma, virtual reality, contact-based educational intervention, virtual patient

## Abstract

**Background:**

Public stigma against depression contributes to low employment rates among individuals with depression. Contact-based educational (CBE) interventions have been shown to reduce this public stigma.

**Objective:**

We investigated the ability of our Virtual Reality Antistigma (VRAS) app developed for CBE interventions to reduce the stigma of depression.

**Methods:**

Sixteen medical students were recruited and randomized 1:1 to the intervention group, who used the VRAS app (VRAS group), and the control group, who watched a video on depression. The depression stigma score was assessed using the Depression Stigma Scale (DSS) and Attitudinal Social Distance (ASD) questionnaire at pre- and postintervention. Feasibility was assessed in both groups and usability was assessed only in the VRAS group after the intervention. A qualitative study was performed on the acquisition of knowledge about stigma in both groups based on participants’ answers to open-ended questions and interviews after the intervention.

**Results:**

The feasibility score was significantly higher in the VRAS group (mean 5.63, SD 0.74) than in the control group (mean 3.88, SD 1.73; *P*=.03). However, no significant differences were apparent between the VRAS and control groups for the DSS (VRAS: mean 35.13, SD 5.30; control: mean 35.38, SD 4.50; *P*=.92) or ASD (VRAS: mean 12.25, SD 3.33; control: mean 11.25, SD 1.91; *P*=.92). Stigma scores tended to decrease; however, the stigma-reducing effects of the VRAS app were not significant for the DSS (pre: mean 33.00, SD 4.44; post: mean 35.13, SD 5.30; *P*=.12) or ASD (pre: mean 13.25, SD 3.92; post: mean 12.25, SD 3.33; *P*=.12). Qualitative analysis suggested that the VRAS app facilitated perspective-taking and promoted empathy toward the patient.

**Conclusions:**

The CBE intervention using virtual reality technology (VRAS app) was as effective as the video intervention. The results of the qualitative study suggested that the virtual reality intervention was able to promote perspective-taking and empathy toward patients.

**Trial Registration:**

University Hospital Medical Information Network Clinical Trials Registry (UMIN-CTR) UMIN000043020; https://upload.umin.ac.jp/cgi-open-bin/ctr/ctr_view.cgi?recptno=R000049109

## Introduction

Major depressive disorder (depression) is a severe medical illness accounting for more than 15 million disability-adjusted life years [1]. Individuals with depression must deal with the illness itself and the stigma (misconceptions) associated with depression [2]. One of the causes of stigma is a lack of knowledge about depression [3]. Stigmatization by the public, reflected in attitudes such as “people complaining of depression are weak,” is known as public stigma [3], whereas internalizing such public stigma and viewing oneself as “weak” (self-devaluation) is known as self-stigma [4,5]. Public stigma contributes to a low employment rate of patients with depression and self-stigmatization by patients [2]. Self-stigma impedes help-seeking [6]; thus, reducing public stigma toward patients with depression is crucial. Like the general public, health care providers may also stigmatize patients, such as viewing depression as a sign of personal weakness. Such stigma can consequently affect the quality of care offered to patients [7,8]. Health care providers thus need to be targeted when addressing issues of stigma surrounding depression.

Many interventions have been conducted to reduce the public stigma of mental illness [7,9,10]. Among these, contact-based educational (CBE) interventions have been shown to reduce stigma against mental illness [10,11]. A CBE intervention, which is based on the intergroup contact theory, uses contact with the stigmatized group to provide knowledge, reduce anxiety, and enhance perspective-taking and empathy toward the stigmatized group to reduce stigma [12].

Two main avenues are available for a CBE intervention: in-person [11] and video-based contact, which is a typical media-based contact [13]. In-person contact requires an actual patient who provides their testimony in a presentation [11,14,15]. However, in-person contact has been conducted less frequently than video-based contact [10] because of the difficulties in recruiting patients [16] and the negative effects of identity disclosure. Moreover, the intervention requires well-trained individuals with a history of mental illness to deliver the presentation [17]. By contrast, video-based contact uses premade videos of patients providing testimonies about their illness [13,18], which is much easier to set up and is more cost-effective to disseminate [19].

In recent years, virtual reality (VR) has been used for intergroup contact, promoting perspective-taking and empathy [20-22]. The usage of VR for intergroup contact also allows researchers to measure and monitor changes in the intergroup interactions in real time [23]. VR has also increasingly been used in the field of mental health [24,25] to reduce anxiety, such as interaction with a virtual spider to help treat arachnophobia (fear of spiders) [26] and interaction with virtual humans to treat social anxiety disorder [27]. In addition, VR has been used to reduce the public stigma of schizophrenia [28-30].

Perspective-taking allows the user to experience a situation from the perspective of another individual, leading to a better understanding of others, affecting others’ evaluations, and enhancing empathy [31,32]. Therefore, it was expected that a CBE intervention using VR for depression stigma may lead to improved knowledge of depression, reduction in anxiety, and an increase in empathy, resulting in an overall reduction in stigma [12].

In this study, we investigated the ability of the Virtual Reality Antistigma (VRAS) app, which was developed for CBE interventions, to reduce the stigma of depression.

## Methods

### Participants

Participants were recruited online. The study was performed in August 2019. Participants were recruited from the University of Tokyo Graduate School of Medicine and School of Medicine. They were informed about the title and background of the study through the website. Exclusion criteria included a current diagnosis of depression or having a history of depression. No payment or reward was offered or provided for participation in the trial. The trial was completed within 1 month of recruitment.

### VRAS App

The VRAS app was developed for Android OS smartphones (Google LLC, Mountain View, CA) to be used with a head-mounted display (HMD). The duration of the VRAS experience was approximately 5 minutes, and the frame rate was set to 60 frames per second to reduce the risk of VR sickness [33].

**Figure 1:**
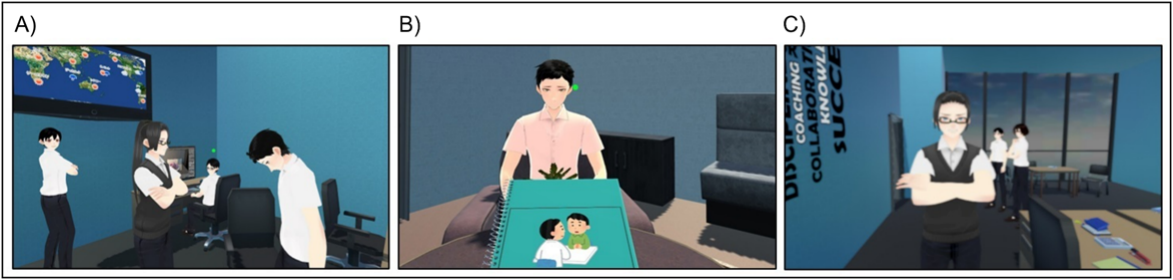
The three sections of the Virtual Reality Antistigma (VRAS) app. (A) User watches the patient being scolded by the boss due to depression stigma. (B) User takes on the role of the counselor in listening to testimonies by the patient. (C) User, as a patient, is scolded directly by the boss.

The VRAS app provides an immersive virtual environment consisting of three sections (Figure 1). The first section was a workplace environment experienced from a third-person perspective, in which the user observed a scene where the patient was scolded by his boss who stigmatized the patient. The purpose was to improve understanding toward depression stigma in a workplace setting from the perspective of a colleague. The second section was a counseling environment experienced from a second-person perspective. The user took on the role of a counselor to ask three questions related to the effect of depression on the life of the patient. The purpose was to educate the user about the symptoms of depression and struggles of the patient. The third section used the first-person perspective; the user assumed the role of the patient and experienced the symptoms of depression and workplace stigma, with the purpose of inducing empathy [34,35].

The VRAS app thus allowed users to interact with a virtual patient with depression from different perspectives. The virtual patient provided testimony about his struggles against depression, as an essential component of CBE interventions [17,36]. Educational messages for the VRAS app include (1) messages about the high prevalence of mental disorders, (2) messages about social inclusion/human rights, and (3) messages emphasizing that the patient is also a human being [37]. All three of these message types have been demonstrated to be helpful in reducing stigma [13,38].

### Video Material

Participants in the control group watched the video “I Had a Black Dog,” written and illustrated by Matthew Johnstone in collaboration with the World Health Organization. The video served as the CBE material, because it showed an animation of a virtual patient, included testimony from the patient about his struggle with depression [17,36], and replaced false beliefs about depression with correct information (“myth-busting”) [17,39]. The video contained educational messages about (1) the high prevalence of mental disorders, (2) social inclusion/human rights, and (3) recovery-oriented practices [37]. These messages have been demonstrated to be effective in reducing stigma [13,38]. This video was of similar duration to the VRAS app (about 5 minutes). The Japanese translation of the official Japanese picture book “I Had a Black Dog” was used as the subtitle for the video.

### Trial Design

**Figure 2:**
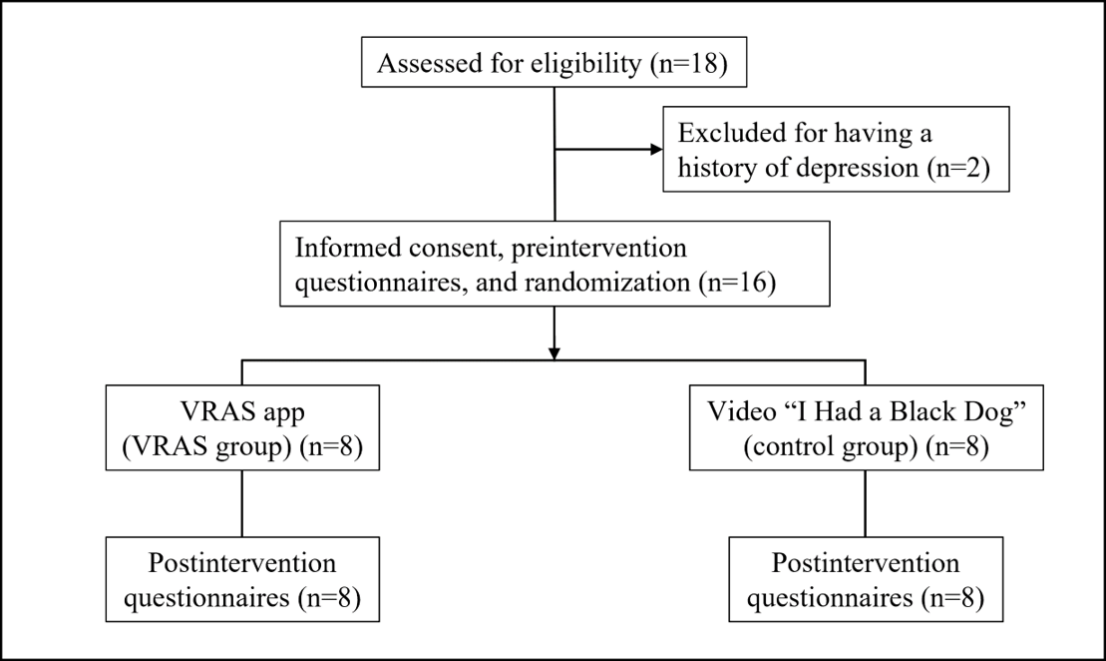
Flowchart of the study. VRAS: Virtual Reality Antistigma.

This was a randomized, controlled pilot study combined with a qualitative study to compare the effect of the VRAS app and video materials on depression. Participants were randomized 1:1 to the intervention group (VRAS group) or control group (Figure 2). Randomization was performed by randomly allocating a number from 0 to 1 to each participant using Microsoft Excel software. Randomization, enrollment, and assignment of participants were performed by the main experimenter overseeing the trial. Neither the participants nor experimenters were blinded in this study.

By referring to a previous study using similar scales [40], the sample size was calculated to allow performance of an independent-samples *t* test with a significance level (α) of 5%, power of 80%, mean difference of 0.75, and standard deviation of 0.5. The number of participants required was calculated as eight per group.

The flow of the trial was as follows. The participant first provided informed consent, completed a set of preintervention questionnaires (participant’s knowledge of depression and stigma, Depression Stigma Scale [DSS], and Attitudinal Social Distance [ASD]), and was randomized to the VRAS group or control group. Participants in the VRAS group used the VRAS app, whereas those in the control group watched the video material. After using the VRAS app or watching the video, the participant immediately completed a set of postintervention questionnaires (DSS, ASD, and feasibility scale for both groups, and a usability scale for the VRAS group only). Participants were required to complete all three sections of the VRAS app. No changes were made to the VRAS app between recruitment and the end of the trial period. The trial was conducted face-to-face with one participant at a time, and only once per participant. The duration of the trial was 30 minutes. Participants in the VRAS group were instructed to stop immediately if they experienced any VR sickness during the VR experience.

### Outcomes

The preintervention survey measured participants’ knowledge of depression and public stigma, as well as the degree of stigma. To measure the degree of public stigma, we used the following vignette of an individual with depression (the Japanese version was used for this study) [41,42]:

John is 30 years old. He has been feeling unusually sad and miserable for the last few weeks. Even though he is tired all the time, he has trouble sleeping nearly every night. John doesn’t feel like eating and has lost weight. He can’t keep his mind on his work and puts off making decisions. Even day-to-day tasks seem too much for him. This has come to the attention of his boss, who is concerned about John’s lowered productivity.

The DSS was used for participants to describe the individual in the vignette along a 5-point Likert scale; a higher DSS score indicates less stigmatization [40,41]. The ASD (also on a 5-point Likert scale) was used to measure the willingness of participants to have contact with the individual in the vignette; a lower ASD score indicates greater willingness [43].

A feasibility scale was also used to assess the feasibility of the VRAS app and video material as an educational tool. A usability scale was used to evaluate the usability of the VRAS app. Both the feasibility and usability scales were modified from the Web-Based Learning Tool (WBLT) [44] and used a 7-point Likert scale (1=strongly disagree, 4=agree, 7=strongly agree).

### Statistical Analysis

A *t* test was applied to the pre- and postintervention results for within- and between-group comparisons. Data were analyzed using the Python programming language (Python 3.6.9; Python Software Foundation) [45], with the significance level set at α=.05.

### Qualitative Study

Open-ended questions were provided for participants to answer after the intervention. All questionnaires were self-administered. Participants in both the VRAS and control groups were asked to describe what they understood about stigma against depression after their experience and were told to write “none” if they still did not understand stigma after the intervention. Participants in the VRAS group were asked about their overall impressions on the features of the VRAS app and if there were any features in need of improvement. Participants in the control group were given an opportunity to experience the VRAS app after the experiment and were then interviewed regarding the VRAS app and video material.

The results of the qualitative studies were analyzed using MAXQDA2022 software (VERBI Software, Berlin, Germany) and were coded by two authors (HO and LW) based on the mediators of intergroup contact: “Knowledge acquisition,” “Anxiety reduction,” “Perspective-taking,” and “Empathy” [12].

### Ethical Considerations

The study is registered at the University Hospital Medical Information Network Clinical Trials Registry (UMIN-CTR; UMIN000043020). Trial registration was completed retrospectively because ethics approval was obtained from the Research Ethics Committee of The University of Tokyo (approval number 2019099NI) before conducting the trial.

## Results

### Participant Characteristics

Eighteen students volunteered for this study and disclosed their medical history of depression. Two volunteers were excluded from the study due to a history of depression. The remaining 16 students completed the trial and their data were analyzed. The characteristics of participants at baseline are provided in Table 1. Before the intervention, 50% of participants in the VRAS group and 63% of participants in the control group showed some degree of knowledge about stigma against depression (Table 1). All participants in both groups completed the trial (VRAS app and video).

**Table 1:** Participant characteristics at baseline (N=16).

Characteristics		Total sample (N=16), n (%)	VRAS^a^ group (n=8), n (%)	Control group (n=8), n (%)	*P* value	
**Sex**						.25
	Male	4 (25)	3 (38)	1 (13)		
	Female	12 (75)	5 (62)	7 (87)		
**Student status**						.30
	Graduate student	15 (94)	7 (88)	8 (100)		
	Undergraduate student	1 (6)	1 (12)	0 (0)		
**Knowledge about depression as a medical illness**						>.99
	Yes	16 (100)	8 (100)	8 (100)		
	No	0	0	0		
**Contact with a patient with depression**						.52
	Yes	13 (81)	6 (75)	7 (88)		
	No	3 (19)	2 (25)	1 (12)		
**Have received education on depression**						.11
	Yes	11 (69)	4 (50)	7 (88)		
	No	5 (31)	4 (50)	1 (12)		
**Knowledge about stigma against depression**						.61
	Yes	9 (56)	4 (50)	5 (63)		
	No	7 (44)	4 (50)	3 (37)		

^a^VRAS: Virtual Reality Antistigma app.

### DSS Scores

No significant differences between the intervention and control groups were identified for the nine DSS items preintervention (*P*=.81) or postintervention (*P*=.92) (Table 2). In addition, none of the nine items differed significantly between pre- and postintervention. After the intervention, the mean scores for both the VRAS and control groups tended to increase (suggesting decreased stigma); however, these differences were not significant (*P*=.12 and *P*=.15, respectively) (Table 2). For the VRAS group, a significant decrease in stigma was seen only for the item “People with this problem are unpredictable” (*P*=.01). In the control group, no mean score was significantly different before and after the intervention.

**Table 2:** Detailed Depression Stigma Scale scores pre- and postintervention.

Item	VRAS^a^ group (n=8)				Control group (n=8)		
	Pre, mean (SD)	Post, mean (SD)	*P* value^b^	Pre, mean (SD)		Post, mean (SD)	*P* value^b^
1. Person could snap out of the problem	4.00 (1.07)	4.38 (1.06)	.08	4.00 (0.76)		4.13 (0.83)	.69
2. Problem is a sign of personal weakness	4.38 (0.74)	4.50 (0.76)	.60	4.00 (0.76)		4.25 (0.89)	.56
3. Problem is not a real medical illness	4.25 (0.71)	4.38 (0.74)	.35	4.25 (0.89)		4.38 (0.92)	.60
4. People with this problem are dangerous	4.00 (0.76)	4.38 (0.52)	.29	3.38 (0.52)		4.00 (0.76)	.14
5. Avoid people with this problem	4.25 (0.71)	4.25 (0.71)	1.00	4.13 (0.99)		4.38 (0.74)	.52
6. People with this problem are unpredictable	3.25 (0.46)	4.13 (0.64)	.01	3.38 (0.52)		3.50 (0.76)	.69
7. If I had this problem, I would not tell anyone	3.13 (0.99)	2.88 (1.25)	.45	3.25 (0.89)		3.63 (0.52)	.08
8. I would not employ someone with this problem	3.25 (1.04)	3.25 (1.04)	>.99	3.63 (0.74)		3.63 (0.74)	>.99
9. I would not vote for a politician with this problem	2.50 (1.20)	3.00 (1.20)	.17	3.50 (1.07)		3.50 (1.07)	>.99
Total personal stigma	33.00 (4.44)	35.13 (5.30)	.12	33.50 (3.46)		35.38 (4.50)	.15

^a^VRAS: Virtual Reality Antistigma app.

^b^Two-tailed paired *t* test.

### ASD Scores

No significant difference in the mean total ASD score between groups was evident preintervention (*P*=.49) or postintervention (*P*=.47) (Table 3). None of the five items in ASD differed significantly from pre- to postintervention for either group. Tendencies toward a decrease in the mean total score, suggesting increased willingness for contact, were seen in both the VRAS and control groups, but the differences were not significant (*P*=.21 and *P*=.11, respectively) (Table 3). For the VRAS group, only the score for the item “Make friends with the person” showed a significant increase in willingness after the intervention (*P*=.03). In the control group, no items showed significant differences in mean scores before and after the intervention.

**Table 3:** Attitudinal Social Distance scores pre- and postintervention.

Item	VRAS^a^ group (n=8)				Control group (n=8)		
	Pre, mean (SD)	Post, mean (SD)	*P* value^b^	Pre, mean (SD)		Post, mean (SD)	*P* value^b^
1. Move next door to the person	2.88 (0.83)	2.75 (0.89)	.60	2.75 (0.71)		2.38 (0.52)	.20
2. Spend an evening socializing with the person	2.38 (0.92)	2.25 (0.89)	.60	2.13 (0.64)		2.00 (0.53)	.35
3. Make friends with the person	2.63 (0.74)	2.13 (0.64)	.03	2.25 (0.46)		2.38 (0.52)	.35
4. Work closely on a job with the person	2.50 (0.93)	2.50 (0.76)	>.99	2.38 (0.74)		2.00 (0.53)	.08
5. Have the person marry into the family	2.88 (0.99)	2.63 (0.74)	.17	2.63 (0.52)		2.50 (0.53)	.35
Total score	13.25 (3.92)	12.25 (3.33)	.21	12.13 (2.03)		11.25 (1.91)	.11

^a^VRAS: Virtual Reality Antistigma app.

^b^Two-tailed paired *t* test.

### Feasibility Scale

The perceived feasibility of the interventions was compared between the two groups (Table 4). No significant difference was seen for the items “This intervention provides useful knowledge related to stigma” or “I think this intervention is an appropriate educational tool to learn about depression stigma” between groups (*P*=.17 and *P*=.26, respectively). However, a significant difference in score was identified for “I understood depression stigma through this intervention,” suggesting that participants in the VRAS group had gained a better understanding of stigma against depression postintervention compared to the control group.

**Table 4:** Detailed scores of the feasibility scale.

Questions	VRAS^a^ group (n=8), mean (SD)	Control group (n=8), mean (SD)	*P* value^b^
1. This intervention provides useful knowledge related to stigma	5.38 (1.06)	4.50 (1.31)	.17
2. I think this intervention is an appropriate educational tool to learn about depression stigma	5.50 (1.31)	4.50 (2.00)	.26
3. I understood depression stigma through this intervention	5.63 (0.74)	3.88 (1.73)	.03

^a^VRAS: Virtual Reality Antistigma app.

^b^Two-tailed unpaired t-test.

### Usability Scale

Based on a score range of 1 to 7, the mean scores for the items “This VR experience is physically stressful” and “This VR experience is mentally stressful” were 3.13 (SD 1.73) and 2.75 (SD 1.58), respectively, suggesting that users did not find the VRAS experience to be particularly stressful. Participants also did not find the VR experience to be “long” (mean 1.75, SD 0.71) or “boring” (mean 1.75, SD 0.71). The mean score for “I had VR sickness during this VR experience” was 1.63 (SD 1.19), suggesting that users did not experience severe VR sickness using the VRAS app. No technical issues with the VRAS app were reported, and all participants managed to complete the VR experience without any difficulties.

In terms of feedback on features of the VRAS app in need of improvement, participants commented that they hoped to learn about methods to better deal with individuals suffering from depression, a refresher section to reinforce the knowledge gained, more scenarios emphasizing recovery, and scenarios involving family members of the patient.

As for the overall impression of the VRAS app, the following feedback was received from participants: “The VRAS application contains good educational content,” “The usage of perspective-taking from a third-person to a first-person perspective provided a better understanding of depression stigma,” “The scenario seems exaggerated and might not happen in a real-life situation,” “I experienced some delay in movement in the virtual world,” and “I think that the VRAS application would be useful as a training tool for managers.”

### Qualitative Analysis

The total number of comments in the VRAS group (n=8) was 33 and that in the control group (n=8) was 6. Three participants in the control group commented “none” related to their understanding of stigma. In the VRAS group, 14 sentences were related to knowledge acquisition, 4 sentences were related to perspective-taking, and 2 sentences were related to empathy. In the control group, 5 sentences were related to knowledge acquisition and 1 sentence was related to empathy; however, there was no comment related to perspective-taking. In both groups, none of the comments was related to anxiety reduction. The other comments in the VRAS group were related to the usability of the VRAS app.

All eight participants in the control group were interviewed on the VRAS experience after the video experience, with two sentences pertaining to knowledge acquisition and one sentence each pertaining to perspective-taking and empathy, respectively. There was no sentence on anxiety reduction.

Typical comments regarding knowledge acquisition in the VRAS group included “I learned that colleagues and superiors discriminate against patients with depression by viewing them as lazy and weak despite being sick,” “Stigma worsens depression,” and “A lack of knowledge causes stigma.” A typical comment indicating a shift in perspective was “I think I was able to understand the patient with depression better as I was able to experience third, second perspective, and first-person perspectives.” Typical comments on empathy included “I understood people with depression from many angles,” “I can understand the patient from many different perspectives,” and “I realized that the stigma of depression makes it difficult for patients to have their symptoms understood by those around them, and it also makes them feel bad, which worsens their depression.”

In the interviews conducted with the participants in the control group after testing the VRAS app, the following comments were made: “I can understand the patient’s feelings in the VR environment,” “The VR experience was useful to understand the patient’s story,” “VR is better than video because it is more immersive,” and “I can concentrate better in VR.”

## Discussion

### Principal Results

We compared the effect of the VRAS app developed for CBE interventions with that of a video intervention on reducing stigma related to depression. CBE using the VRAS intervention was as effective as the video intervention. The qualitative study suggested that the VRAS intervention generated more empathy for patients with depression by shifting users’ perspectives to that of the patient.

In the following, the results are discussed in accordance with the mediators of intergroup contact for reducing public stigma mentioned above (ie, knowledge acquisition, anxiety reduction, perspective-taking, and empathy).

### Knowledge Acquisition

Although there was a decrease in stigma after the intervention in the VRAS group, the difference was not statistically significant. We speculate that this may be due to the educational content of the VRAS app. As the VR experience was limited to approximately 5 minutes to reduce the risk of VR sickness [33], we included only educational messages designed to reduce stigma [13,38], such as messages about the high prevalence of mental illness and social inclusion/human rights, as well as “see the person” messages [37]. A recent study also showed that the inclusion of biomedical content (eg, the biological mechanisms underlying mental illness, including neurotransmitters such as dopamine and serotonin) could reduce stigma [46]. Thus, the knowledge gained from the VRAS experience may have been insufficient to reduce depression stigma.

To ensure that the participants had a similar background and knowledge of depression, only medical students were enrolled in this study. Biomedical content may have been particularly relevant to improve the effectiveness of the VRAS app in this population. The lack of such content may have affected the stigma scores. However, the optimal educational content to reduce stigma is controversial [47]. In any case, providing knowledge appropriate to the participant is essential [48]. We believe that VR technology can accomplish this, because it enables cost-effective modification of educational content to fit the demographic characteristics of the user.

The results of the qualitative study suggested that the VRAS group acquired knowledge about stigma more readily than the control group. This may be attributed to the fact that the VRAS app deepened the user’s understanding of stigma by allowing them to meet the virtual patient in a virtual world and experience their behavior and speech in environments similar to the real world. In addition, the participants in the VRAS group commented that the different perspectives of the VRAS app enhanced their understanding of stigma, which was reflected by the results of superior knowledge acquisition in the VRAS group. The usage of the VRAS app for the CBE intervention also overcame some of the difficulties such as ethical issues associated with face-to-face interactions with patients.

### Anxiety Reduction

None of the comments from the participants indicated a reduction in anxiety. A prior study using VR to reduce the stigma of schizophrenia showed a reduction in stigma only for participants who liked the person encountered, suggesting that a more positive evaluation of the virtual patient may lead to a reduction in anxiety [28]. However, because schizophrenia and depression have different symptoms, we did not measure the anxiety level of the participants.

### Perspective-taking and Empathy

An advantage of VR interventions is the incorporation of perspective-taking to stimulate empathy. Perspective-taking and empathy have been suggested to be helpful in reducing stigma toward others [12,31,32]. The VRAS app utilized a third-person perspective to provide a short example of stigma, a second-person perspective to allow the user to learn about the struggles of patients with depression, and a first-person perspective to put the user “in the shoes” of a patient to understand the experience of having depression and being stigmatized. From the results of the qualitative analysis, it was suggested that the VRAS experience was able to induce empathy and perspective-taking in the participants. However, the total task duration of 5 minutes may have been insufficient to enhance the empathy required, as reflected by the nonsignificant reduction in stigma. Although the optimal duration of intergroup contact is contentious and requires further research [23], VR is immersive and may be a suitable tool to assist in inducing empathy in a short duration.

### Usability of the VRAS App

Hardware issues also affected the results of this study. The high latency in the virtual world may have negatively affected knowledge acquisition, undermining the effectiveness of the VRAS app. In the qualitative study, some participants commented that they experienced a delay in movement in the virtual world. Latency is defined as the delay before data transfer begins after an instruction to enact transfer. A latency of >50 milliseconds has been reported to lead to unpleasant VR experiences by reducing the sense of presence in the virtual world [49]. This may have undermined the learning effectiveness of the VRAS app [50]. The VRAS app was developed for use with Android OS smartphones and an HMD; however, smartphones are generally inadequate for VR apps because of latency issues [51]. The VRAS app had an average latency of approximately 70 milliseconds, which may have degraded the user experience and negatively impacted learning [52].

All participants managed to complete the entire VR task without any issue. Although the VRAS app had high latency, we improved its usability by restricting the duration to about 5 minutes to reduce the risk of VR sickness. Frame rate has been shown to affect performance more than latency [53]. We therefore developed the VRAS app to run at approximately 60 frames per second, which is considered sufficient [33].

Regarding the usability of the VRAS app, most participants did not find the VR experience to be physically or mentally stressful, or overly long, and none of the participants experienced VR sickness. Participants also did not find the VR experience to be boring or tedious. This may be due to the scenario design of the VRAS app in which participants interacted with the patient in different roles through the three sections. Interaction within a virtual world has been shown to improve user engagement with the learning materials and to make learning more enjoyable [54].

### Limitations

To our knowledge, this is the first randomized controlled pilot study combined with a qualitative study aiming to reduce the stigma of depression via a CBE intervention using VR technology compared to a video intervention. This study had several limitations. First, the participants and instructor were not blinded, which may have affected motivation. Second, the sample size was small; thus, further, larger studies are needed to validate the effectiveness of the VRAS app. Third, the baseline level of depression stigma in this study was low; therefore, the app should be tested in a population with a higher level of stigma. Fourth, the VRAS app focuses on the workplace environment; scenarios involving environments such as schools and the home are needed to expand its scope. Fifth, the duration and content of the video materials were similar, but not identical, to those of the VRAS app. Therefore, the validity of the video control needs to be examined. Sixth, because no suitable feasibility scale for this intervention type has been reported, we adapted items from the WBLT rating scale to create our scale, which needs to be validated in further studies.

### Conclusions

The CBE intervention using VR technology (VRAS app) was as effective as a video intervention. The qualitative study suggested that the VR intervention was able to enhance empathy for patients, attributed to the perspective-taking. Further research with a larger number of participants is warranted.

## Appendix

Multimedia Appendix 1 CONSORT-eHEALTH checklist (V 1.6.1).

## Abbreviations

ASD: Attitudinal Social Distance scale
CBE: contact-based education
DSS: Depression Stigma Scale
HMD: head-mounted display
VR: virtual reality
VRAS: Virtual Reality Antistigma app
WBLT: Web-Based Learning Tool

## References

[1] Murray CJL, Vos T, Lozano R, Naghavi M, Flaxman AD, Michaud C, Ezzati M, Shibuya K, Salomon JA, Abdalla S, Aboyans V, Abraham J, Ackerman I, Aggarwal R, Ahn SY, Ali MK, Alvarado M, Anderson HR, Anderson HR, Andrews KG, Atkinson C, Baddour LM, Bahalim AN, Barker-Collo S, Barrero LH, Bartels DH, Basáñez MG, Baxter A, Bell ML, Benjamin EJ, Bennett D, Bernabé E, Bhalla K, Bhandari B, Bikbov B, Bin Abdulhak A, Birbeck G, Black JA, Blencowe H, Blore JD, Blyth F, Bolliger I, Bonaventure A, Boufous S, Bourne R, Boussinesq M, Braithwaite T, Brayne C, Bridgett L, Brooker S, Brooks P, Brugha TS, Bryan-Hancock C, Bucello C, Buchbinder R, Buckle G, Budke CM, Burch M, Burney P, Burstein R, Calabria B, Campbell B, Canter CE, Carabin H, Carapetis J, Carmona L, Cella C, Charlson F, Chen H, Cheng AT, Chou D, Chugh SS, Coffeng LE, Colan SD, Colquhoun S, Colson KE, Condon J, Connor MD, Cooper LT, Corriere M, Cortinovis M, de Vaccaro KC, Couser W, Cowie BC, Criqui MH, Cross M, Dabhadkar KC, Dahiya M, Dahodwala N, Damsere-Derry J, Danaei G, Davis A, De Leo D, Degenhardt L, Dellavalle R, Delossantos A, Denenberg J, Derrett S, Des Jarlais DC, Dharmaratne SD, Dherani M, Diaz-Torne C, Dolk H, Dorsey ER, Driscoll T, Duber H, Ebel B, Edmond K, Elbaz A, Ali SE, Erskine H, Erwin PJ, Espindola P, Ewoigbokhan SE, Farzadfar F, Feigin V, Felson DT, Ferrari A, Ferri CP, Fèvre EM, Finucane MM, Flaxman S, Flood L, Foreman K, Forouzanfar MH, Fowkes FGR, Fransen M, Freeman MK, Gabbe BJ, Gabriel SE, Gakidou E, Ganatra HA, Garcia B, Gaspari F, Gillum RF, Gmel G, Gonzalez-Medina D, Gosselin R, Grainger R, Grant B, Groeger J, Guillemin F, Gunnell D, Gupta R, Haagsma J, Hagan H, Halasa YA, Hall W, Haring D, Haro JM, Harrison JE, Havmoeller R, Hay RJ, Higashi H, Hill C, Hoen B, Hoffman H, Hotez PJ, Hoy D, Huang JJ, Ibeanusi SE, Jacobsen KH, James SL, Jarvis D, Jasrasaria R, Jayaraman S, Johns N, Jonas JB, Karthikeyan G, Kassebaum N, Kawakami N, Keren A, Khoo JP, King CH, Knowlton LM, Kobusingye O, Koranteng A, Krishnamurthi R, Laden F, Lalloo R, Laslett LL, Lathlean T, Leasher JL, Lee YY, Leigh J, Levinson D, Lim SS, Limb E, Lin JK, Lipnick M, Lipshultz SE, Liu W, Loane M, Ohno SL, Lyons R, Mabweijano J, MacIntyre MF, Malekzadeh R, Mallinger L, Manivannan S, Marcenes W, March L, Margolis DJ, Marks GB, Marks GB, Matsumori A, Matzopoulos R, Mayosi BM, McAnulty JH, McDermott MM, McGill N, McGrath J, Medina-Mora ME, Meltzer M, Mensah GA, Merriman TR, Meyer AC, Miglioli V, Miller M, Miller TR, Mitchell PB, Mock C, Mocumbi AO, Moffitt TE, Mokdad AA, Monasta L, Montico M, Moradi-Lakeh M, Moran A, Morawska L, Mori R, Murdoch ME, Mwaniki MK, Naidoo K, Nair MN, Naldi L, Narayan KMV, Nelson PK, Nelson RG, Nevitt MC, Newton CR, Nolte S, Norman P, Norman R, O'Donnell M, O'Hanlon S, Olives C, Omer SB, Ortblad K, Osborne R, Ozgediz D, Page A, Pahari B, Pandian JD, Rivero AP, Patten SB, Pearce N, Padilla RP, Perez-Ruiz F, Perico N, Pesudovs K, Phillips D, Phillips MR, Pierce K, Pion S, Polanczyk GV, Polinder S, Pope CA, Popova S, Porrini E, Pourmalek F, Prince M, Pullan RL, Ramaiah KD, Ranganathan D, Razavi H, Regan M, Rehm JT, Rein DB, Remuzzi G, Richardson K, Rivara FP, Roberts T, Robinson C, De Leòn FR, Ronfani L, Room R, Rosenfeld LC, Rushton L, Sacco RL, Saha S, Sampson U, Sanchez-Riera L, Sanman E, Schwebel DC, Scott JG, Segui-Gomez M, Shahraz S, Shepard DS, Shin H, Shivakoti R, Singh D, Singh GM, Singh JA, Singleton J, Sleet DA, Sliwa K, Smith E, Smith JL, Stapelberg NJC, Steer A, Steiner T, Stolk WA, Stovner LJ, Sudfeld C, Syed S, Tamburlini G, Tavakkoli M, Taylor HR, Taylor JA, Taylor WJ, Thomas B, Thomson WM, Thurston GD, Tleyjeh IM, Tonelli M, Towbin JA, Truelsen T, Tsilimbaris MK, Ubeda C, Undurraga EA, van der Werf MJ, van Os J, Vavilala MS, Venketasubramanian N, Wang M, Wang W, Watt K, Weatherall DJ, Weinstock MA, Weintraub R, Weisskopf MG, Weissman MM, White RA, Whiteford H, Wiebe N, Wiersma ST, Wilkinson JD, Williams HC, Williams SRM, Witt E, Wolfe F, Woolf AD, Wulf S, Yeh PH, Zaidi AKM, Zheng ZJ, Zonies D, Lopez AD, AlMazroa MA, Memish ZA (2012). Disability-adjusted life years (DALYs) for 291 diseases and injuries in 21 regions, 1990-2010: a systematic analysis for the Global Burden of Disease Study 2010. Lancet.

[2] Stuart H (2004). Stigma and work. Healthc Pap.

[3] Ando S, Yamaguchi S, Aoki Y, Thornicroft G (2013). Review of mental-health-related stigma in Japan. Psychiatry Clin Neurosci.

[4] Corrigan P (2004). How stigma interferes with mental health care. Am Psychol.

[5] Corrigan PW, Rao D (2012). On the self-stigma of mental illness: stages, disclosure, and strategies for change. Can J Psychiatry.

[6] Barney LJ, Griffiths KM, Jorm AF, Christensen H (2006). Stigma about depression and its impact on help-seeking intentions. Aust N Z J Psychiatry.

[7] Finkelstein J, Lapshin O (2007). Reducing depression stigma using a web-based program. Int J Med Inform.

[8] Vankar JR, Prabhakaran A, Sharma H (2014). Depression and stigma in medical students at a private medical college. Indian J Psychol Med.

[9] Corrigan PW, River LP, Lundin RK, Penn DL, Uphoff-Wasowski K, Campion J, Mathisen J, Gagnon C, Bergman M, Goldstein H, Kubiak MA (2001). Three strategies for changing attributions about severe mental illness. Schizophr Bull.

[10] Griffiths KM, Carron-Arthur B, Parsons A, Reid R (2014). Effectiveness of programs for reducing the stigma associated with mental disorders. A meta-analysis of randomized controlled trials. World Psychiatry.

[11] Stuart H, Koller M, Christie R, Pietrus M (2011). Reducing mental health stigma: a case study. Healthc Q.

[12] Pettigrew TF, Tropp LR (2008). How does intergroup contact reduce prejudice? Meta-analytic tests of three mediators. Eur J Soc Psychol.

[13] Koike S, Yamaguchi S, Ojio Y, Ohta K, Shimada T, Watanabe K, Thornicroft G, Ando S (2016). A randomised controlled trial of repeated filmed social contact on reducing mental illness-related stigma in young adults. Epidemiol Psychiatr Sci.

[14] Pinfold V, Toulmin H, Thornicroft G, Huxley P, Farmer P, Graham T (2003). Reducing psychiatric stigma and discrimination: evaluation of educational interventions in UK secondary schools. Br J Psychiatry.

[15] Pinfold V, Huxley P, Thornicroft G, Farmer P, Toulmin H, Graham T (2003). Reducing psychiatric stigma and discrimination--evaluating an educational intervention with the police force in England. Soc Psychiatry Psychiatr Epidemiol.

[16] Millum J, Campbell M, Luna F, Malekzadeh A, Karim QA (2019). Ethical challenges in global health-related stigma research. BMC Med.

[17] Knaak S, Patten S (2016). A grounded theory model for reducing stigma in health professionals in Canada. Acta Psychiatr Scand.

[18] Ng YP, Rashid A, O'Brien F (2017). Determining the effectiveness of a video-based contact intervention in improving attitudes of Penang primary care nurses towards people with mental illness. PLoS One.

[19] Clement S, van Nieuwenhuizen A, Kassam A, Flach C, Lazarus A, de Castro M, McCrone P, Norman I, Thornicroft G (2012). Filmed v. live social contact interventions to reduce stigma: randomised controlled trial. Br J Psychiatry.

[20] Herrera F, Bailenson J, Weisz E, Ogle E, Zaki J (2018). Building long-term empathy: A large-scale comparison of traditional and virtual reality perspective-taking. PLoS One.

[21] Roswell R, Cogburn C, Tocco J, Martinez J, Bangeranye C, Bailenson J, Wright M, Mieres J, Smith L (2020). Cultivating empathy through virtual reality: advancing conversations about racism, inequity, and climate in medicine. Acad Med.

[22] Oh SY, Bailenson J, Weisz E, Zaki J (2016). Virtually old: Embodied perspective taking and the reduction of ageism under threat. Comput Hum Behav.

[23] O'Donnell AW, Friehs M, Bracegirdle C, Zúñiga C, Watt SE, Barlow FK (2021). Technological and analytical advancements in intergroup contact research. J Soc Issues.

[24] Hussain SA, Park T, Yildirim I, Xiang Z, Abbasi F (2018). Virtual-reality videos to relieve depression. Virtual, augmented and mixed reality: applications in health, cultural heritage, and industry.

[25] Wiederhold BK, Gao K, Sulea C, Wiederhold MD (2014). Virtual reality as a distraction technique in chronic pain patients. Cyberpsychol Behav Soc Netw.

[26] Garcia-Palacios A, Hoffman H, Carlin A, Furness T, Botella C (2002). Virtual reality in the treatment of spider phobia: a controlled study. Behav Res Ther.

[27] Kampmann IL, Emmelkamp PMG, Hartanto D, Brinkman W, Zijlstra BJH, Morina N (2016). Exposure to virtual social interactions in the treatment of social anxiety disorder: A randomized controlled trial. Behav Res Ther.

[28] Stelzmann D, Toth R, Schieferdecker D (2021). Can intergroup contact in virtual reality (VR) reduce stigmatization against people with schizophrenia?. J Clin Med.

[29] Cangas AJ, Navarro N, Parra JMA, Ojeda JJ, Cangas D, Piedra JA, Gallego J (2017). Stigma-Stop: a serious game against the stigma toward mental health in educational settings. Front Psychol.

[30] Kalyanaraman S, Penn D, Ivory J, Judge A (2010). The virtual doppelganger: effects of a virtual reality simulator on perceptions of schizophrenia. J Nerv Ment Dis.

[31] Galinsky AD, Ku G, Wang CS (2005). Perspective-taking and self-other Overlap: fostering social bonds and facilitating social coordination. Gr Process Intergr Relations.

[32] Galinsky AD, Moskowitz GB (2000). Perspective-taking: decreasing stereotype expression, stereotype accessibility, and in-group favoritism. J Person Soc Psychol.

[33] Yao R, Heath T, Davies A, Forsyth T, Mitchell N, Hoberman P (2014). Oculus VR Best Practices Guide.

[34] Bertrand P, Guegan J, Robieux L, McCall CA, Zenasni F (2018). Learning empathy through virtual reality: multiple strategies for training empathy-related abilities using body ownership illusions in embodied virtual reality. Front Robot AI.

[35] Schutte NS, Stilinović EJ (2017). Facilitating empathy through virtual reality. Motiv Emot.

[36] Pinfold V, Thornicroft G, Huxley P, Farmer P (2005). Active ingredients in anti-stigma programmes in mental health. Int Rev Psychiatry.

[37] Clement S, Jarrett M, Henderson C, Thornicroft G (2010). Messages to use in population-level campaigns to reduce mental health-related stigma: consensus development study. Epidemiol Psichiatr Soc.

[38] Yamaguchi S, Ojio Y, Ando S, Bernick P, Ohta K, Watanabe K, Thornicroft G, Shiozawa T, Koike S (2019). Long-term effects of filmed social contact or internet-based self-study on mental health-related stigma: a 2-year follow-up of a randomised controlled trial. Soc Psychiatry Psychiatr Epidemiol.

[39] Corrigan PW, Penn DL (1999). Lessons from social psychology on discrediting psychiatric stigma. Am Psychol.

[40] Griffiths KM, Christensen H, Jorm AF, Evans K, Groves C (2004). Effect of web-based depression literacy and cognitive-behavioural therapy interventions on stigmatising attitudes to depression: randomised controlled trial. Br J Psychiatry.

[41] Griffiths KM, Nakane Y, Christensen H, Yoshioka K, Jorm AF, Nakane H (2006). Stigma in response to mental disorders: a comparison of Australia and Japan. BMC Psychiatry.

[42] Hanzawa S, Nakane Y, Yoshioka K, Nakane H (2008). Stigma and social distance toward persons with mental disorders: comparison between persons with schizophrenia. Jpn J Psychiat Rehabil.

[43] Link BG, Phelan JC, Bresnahan M, Stueve A, Pescosolido BA (1999). Public conceptions of mental illness: labels, causes, dangerousness, and social distance. Am J Public Health.

[44] Kay R (2011). Evaluating learning, design, and engagement in web-based learning tools (WBLTs): The WBLT Evaluation Scale. Comput Hum Behav.

[45] Python.

[46] Ojio Y, Yamaguchi S, Ohta K, Ando S, Koike S (2019). Effects of biomedical messages and expert-recommended messages on reducing mental health-related stigma: a randomised controlled trial. Epidemiol Psychiatr Sci.

[47] Rusch LC, Kanter JW, Brondino MJ (2009). A comparison of contextual and biomedical models of stigma reduction for depression with a nonclinical undergraduate sample. J Nerv Ment Dis.

[48] Haghighat R (2001). A unitary theory of stigmatisation: pursuit of self-interest and routes to destigmatisation. Br J Psychiatry.

[49] Meehan M, Razzaque S, Whitton MC, Brooks FP (2003). Effect of latency on presence in stressful virtual environments.

[50] Mikropoulos TA (2006). Presence: a unique characteristic in educational virtual environments. Virtual Reality.

[51] Raaen K, Kjellmo I, Chorianopoulos K, Divitini M, Baalsrud Hauge J, Jaccheri L, Malaka R (2015). Measuring latency in virtual reality systems. Entertainment Computing - ICEC 2015.

[52] Mantovani F, Castelnuovo G, Riva G, Davide F, IJsselsteijn WA (2003). Sense of presence in virtual training: enhancing skills acquisition and transfer of knowledge through learning experience in virtual environments. Being there: Concepts, effects and measurements of user presence in synthetic environments.

[53] Janzen B, Teather R (2014). Is 60 FPS better than 30? The impact of frame rate and latency on moving target selection.

[54] Christopoulos A, Conrad M, Shukla M (2014). Objects, worlds, and students: virtual interaction in education. Educ Res Int.

